# Two rare cancers of the exocrine pancreas: to treat or not to treat like ductal adenocarcinoma?

**DOI:** 10.20517/2394-4722.2022.106

**Published:** 2023-03-07

**Authors:** Nebojsa Skorupan, Shadin Ghabra, J. Alberto Maldonado, Yang Zhang, Christine Alewine

**Affiliations:** 1 Laboratory of Molecular Biology, NCI Center for Cancer Research, Bethesda, MD 20892, USA; 2 Medical Oncology Program, NCI Center for Cancer Research, Bethesda, MD 20892, USA; 3 Surgical Oncology Program, NCI Center for Cancer Research, Bethesda, MD 20892, USA; 4 Medical Research Scholars Program, NCI Center for Cancer Research, Bethesda, MD 20892, USA; 5 Gastrointestinal Pathology, Joint Pathology Center, Silver Spring, MD 20910, USA

**Keywords:** Adenosquamous carcinoma of the pancreas, acinar cell carcinoma of the pancreas, rare exocrine pancreatic cancer

## Abstract

Pancreatic cancer is an aggressive malignancy with increasing incidence. Pancreatic ductal adenocarcinoma (PDAC) accounts for > 90% of pancreatic cancer diagnoses, while other exocrine tumors are much rarer. In this review, we have focused on two rare cancers of the exocrine pancreas: adenosquamous carcinoma of the pancreas (ASCP) and pancreatic acinar cell carcinoma (PACC). The latest findings regarding their cellular and molecular pathology, clinical characteristics, prognosis, and clinical management are discussed. New genetic and transcriptomic data suggest that ASCP is related to or overlaps with the basal transcriptomic subtype of PDAC. These tumors are highly aggressive and driven by activated *KRAS* and *MYC* expression. Clinical outcomes remain poor and effective treatments are limited. PACC has no morphologic or genetic resemblance to PDAC and more favorable outcomes. Early stage PACC patients have improved survival with surgical resection and patients with advanced disease benefit most from platinum- or fluoropyrimidine-containing chemotherapy. Frequency of actionable genetic mutations is high in this disease and case reports suggest good outcomes when matched therapy is given. Dedicated clinical studies examining ASCP and PACC are limited and difficult to accrue. Further research is needed to define optimal clinical management for these rare diseases.

## INTRODUCTION

Pancreatic cancer is an aggressive malignancy with a 5-year overall survival rate in the United States of just 11% despite recent advances in systemic chemotherapy that have improved outcomes for patients with both advanced and early-stage disease^[1–5]^. While pancreatic cancer contributes only 3.2% of new cancer cases in the US, the high mortality rate has made pancreatic cancer the third most common cause of cancer-related death in the country^[5]^. Since the incidence of pancreas cancer is increasing every year, it is projected to overtake colorectal cancer as the 2nd most common cause of cancer death by 2030^[6]^. Pancreatic cancer has a similarly grim prognosis and incidence trajectory globally^[7]^.

Most pancreatic cancers arise from ductal and acinar cells involved in the exocrine functions of the organ. Pancreatic ductal adenocarcinoma (PDAC) is the most common histology and represents > 90% of pancreatic cancer cases. It is so common compared to other types of pancreatic cancer that mention of *pancreatic cancer* can be assumed synonymous with PDAC unless otherwise specified. Tumors arising from endocrine cells of the pancreas represent ~5% of all pancreas cancers^[8]^ are mostly less aggressive than PDAC, and have entirely different standard-of-care treatment paradigms^[9]^. Even less common than pancreatic neuroendocrine tumors are rare tumors of the exocrine pancreas, such as adenosquamous carcinoma, acinar cell carcinoma, mucinous cystic neoplasm, colloid carcinoma, and pancreatoblastoma. These diseases are so rare that treatment paradigms for them are typically extrapolated from PDAC standard of care even though their histology and molecular underpinnings may differ markedly from PDAC.

In this review, we have described what the field presently knows about two rare exocrine cancers of the pancreas: adenosquamous carcinoma and acinar cell carcinoma. We have defined their cellular and molecular pathology, clinical characteristics, and prognosis. Basic and translational studies examining their origins and behavior have been surveyed. Case studies and epidemiologic reports which provide insights into fruitful treatment paradigms have been reviewed. It is important to note that there are no prospective clinical studies reported in the literature that examine any aspect of these diseases. Significant differences between these tumors and PDAC have been highlighted to provide insight into when clinicians should diverge from established PDAC standards of care when treating these patients. In the end, we aimed to identify the important unanswered clinical questions about these diseases, providing a guide for future research that could allow clinicians to offer the first evidence-based advice to patients.

## STANDARD OF CARE TREATMENT FOR PDAC

PDAC typically presents with non-specific symptoms such as back pain, unexplained weight loss, jaundice, GI discomfort or thromboembolism^[10,11]^. Most patients already have distant metastasis (52%) or locoregional disease (30%) at the time of diagnosis. Primary tumors are most commonly located in the pancreatic head^[12]^, while metastases are most often located in the liver, peritoneum and lung.

The staging for PDAC is shown in Table 1. Current standard of care for early-stage disease (Stage I and II, or Stage III that is not T4) is upfront surgical resection followed by adjuvant chemotherapy. There is no appreciable cure rate if chemotherapy is not given^[13]^. Choices of adjuvant chemotherapy include single-agent gemcitabine for those with poorer performance status, gemcitabine in combination with capecitabine, or modified FOLFIRINOX for those with excellent performance status^[2,4,13]^. More than 50% of patients who complete these potentially curative regimens will recur and die of their disease. Currently, neoadjuvant strategies are being evaluated and may prove more beneficial in patients with resectable disease^[14]^. Notably, complete neoadjuvant treatment is considered the standard of care for patients with borderline resectable and locally advanced diseases at many pancreatic cancer centers. The benefit of chemoradiation has not been clearly established, but it is commonly incorporated in neoadjuvant paradigms, especially in cases of borderline resectable or locally advanced disease^[15]^. Locally advanced (Stage III that is T4) and metastatic (Stage IV) PDAC are treated with palliative chemotherapy. Appropriate regimens for fit patients include FOLFIRINOX or gemcitabine/nanoalbumin-bound (nab-) paclitaxel (GnP), which can extend median survival to 11.5 months^[1,16]^. Single-agent gemcitabine can be given to patients with poorer performance status to provide clinical benefit^[17]^. Of note, PDAC is generally unresponsive to immunotherapy^[18,19]^. Markers of response to immune checkpoint inhibitors - high microsatellite instability (MSI-H) and mismatch repair deficiency (dMMR) - occur in less than 2% of PDAC patients^[20,21]^, but even in this small group, responses to immunotherapy are lower when compared to other patients with MSI-H/dMMR solid tumors^[22]^.

The poor response of PDAC to both chemo- and immunotherapies has most often been attributed to the tumor’s unique microenvironment. PDAC typically has a desmoplastic stroma which makes up approximately 70% of the tumor mass, leading to vascular collapse and hypoxia^[23]^. This results in an “immunologically cold” microenvironment that is mainly infiltrated by immunosuppressive myeloid cells such as tumor-associated macrophages (TAMs), myeloid-derived suppressor cells (MDSCs), tumor-associated neutrophils (TANs), and FOXP3+ CD4+ regulatory T (Treg) cells, with exclusion of tumor-restraining effector T cells (reviewed in^[24]^).

The genomic landscape of PDAC is well known. The four most commonly mutated genes are *KRAS, TP53, CDKN2A,* and *SMAD4*, all of which have been considered “undruggable” until recently^[25,26]^. Notably, mutations in *KRAS* are the primary driver of PDAC oncogenesis, occurring in > 90% of patient cases^[27]^. No other mutations occur more frequently than 10%. Around 5%-9% of PDAC tumors exhibit germline or somatic mutations in homologous recombination (HR) repair-related genes such as *BRCA1/2* or *PALB2*^[28,29]^, and these tumors have significant sensitivity to platinum chemotherapy^[30]^. Treatment of these tumors with platinum-containing regimens doubles patient survival^[31–33]^. These patients may also benefit from maintenance treatment with olaparib, a PARP inhibitor^[34]^.

Transcriptomic profiling has revealed two main PDAC subtypes - a less-aggressive, better differentiated *classical* subtype, and a more aggressive *basal* (also called *quasimesenchymal* or *squamous*) subtype^[35–37]^. It is important to note that some tumors fail to fall distinctly into a single transcriptomic category^[38]^, transcriptomic subtype can be heterogeneous across individual tumors^[39,40]^, and transcriptomic subtype may not be static as treatment or environmental factors may select for a particular subtype^[41,42]^.

## ADENOSQUAMOUS CARCINOMA OF THE PANCREAS (ASCP)

### Pathologic characteristics

#### Histology of ASCP

ASCP is a rare exocrine cancer that includes both a glandular and a malignant squamous component. It was first reported by Herxheimer in 1907 as a “cancroide” tumor. Subsequently, other researchers have called it mucoepidermoid carcinoma, adenoacanthoma, and mixed squamous and adenocarcinoma^[43]^. ASCP is defined by the presence of a malignant squamous component in at least 30% of cancer cells in a background of malignant glandular epithelium^[44,45]^. The arbitrary cutoff of 30% has been disputed in the literature^[45,46]^, with opponents noting that the evaluation is subjective and highly dependent on sampling^[47]^, especially when fine-needle aspiration is used^[48]^. This was further substantiated by a retrospective trial showing that the percentage of cells below or above the 30% cutoff did not correlate with different clinical outcomes^[49]^. Conversely, a very high percentage of squamous cell component (> 60%) was associated with worse survival in resectable patients^[50]^.

**Figure 1:**
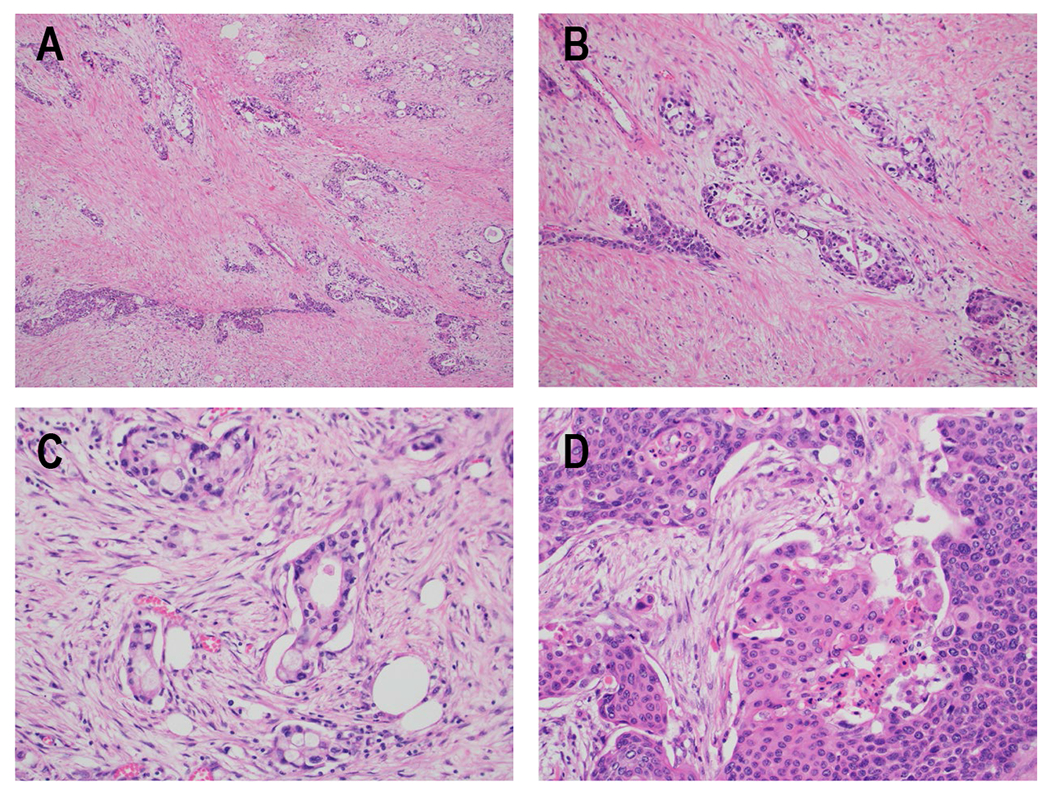
Histologic appearance of ASCP. H&E specimens of ASCP. A) Areas resembling conventional ductal adenocarcinoma of the pancreas (top right) and squamous cell carcinoma (bottom left) are apparent in this sample (100x). Note the striking amount of desmoplastic stroma present between tumor nests. B) In this area of the tumor, the glandular and squamous components have merged together so that both morphologies can be seen within a single group of tumor cells (100x). C) Infiltrative glands making up the adenocarcinoma component of the tumor are shown at higher power (200x), with some cells containing possible mucin vacuoles. D) Solid sheets of tumor cells with densely eosinophilic cytoplasm and areas of keratinization, consistent with the squamous cell carcinoma component of the tumor, are shown at higher power (200x).

Under light microscopy, ASCP is more likely to have increased necrosis, to be poorly differentiated and to have increased vascular invasion compared to PDAC^[51]^. Malignant glandular components of ASCP appear similar to PDAC, while the squamous component has well-defined cell borders with intercellular bridges and keratinization of the cytoplasm^[52]^. Representative H&E-stained images of ASCP are shown in Figure 1. Immunohistochemistry differs between the two components. The glandular component stains positive for CK8/18, CK7, CEA, and CA19-9, similar to PDAC, while appreciable expression of CK5/6, p63, and p40 is seen in the squamous component. The squamous area also exhibits frequent loss of E-cadherin and p16, and increased EGFR and vimentin expression^[52–55]^. Positive nuclear p63 expression has been validated as a more sensitive marker for ASCP in tumors that are difficult to classify by routine H&E stains^[52]^. Others have noted that metastases from an ASCP primary may appear as a pure adenocarcinoma, or even dedifferentiate to giant cell or pleomorphic-appearing anaplastic carcinomas with multi-nucleated forms, markedly bizarre nuclei, and prominent nucleoli^[45]^. Interestingly, a recent study of the transitional area between glandular and squamous components identified an intermediate component that appeared glandular but stained heterogeneously for squamous and glandular markers (i.e., CK8/18 and p63 double-positive)^[55]^. This type of dual staining was also seen in some PDAC samples that lacked any histologic squamous component. Recently, a retrospective study identified a sarcomatous component to ASCP in 7 of 7 cases^[54]^, but this finding has not been mentioned by others except for one case report describing a particularly large ASCP^[56]^. Immunologically, the adenocarcinoma component of ASCP resembles PDAC; however, ASCP has been noted to frequently express PD-L1 within the squamous component^[57,58]^.

#### Molecular profiling of ASCP

Normal pancreatic tissue has no squamous cells, leading to the obvious question of how a squamous component can appear in a primary pancreatic malignancy. Three etiologies were proposed: collision of two separate primaries to form one tumor, metaplasia caused by inflammation resulting in a transition to squamous differentiation in the pre-malignant stage, or trans-differentiation of existing adenocarcinoma to a squamous component^[59]^. Presently, the latter theory is favored given the genomic and transcriptomic data described below, which are consistent with ASCP appearing following activation of squamous differentiation programs in a pre-existing epithelial cancer cell^[35,39]^.

**Figure 2:**
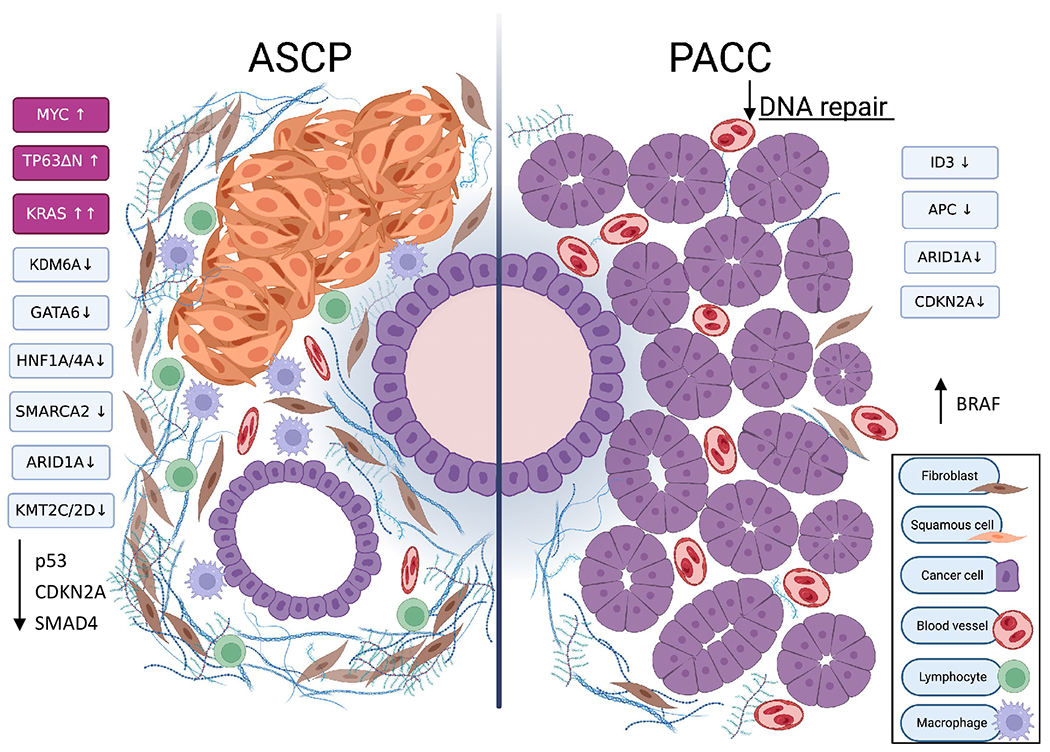
Molecular characteristics of ASCP and PACC *Left*- Adenosquamous carcinoma of the pancreas (ASCP) is characterized by *KRAS, MYC and TP63ΔN* activation. Epigenetic changes cause loss of expression of chromatin modifying genes such as *KDM6A* and *KMT2C/D* as well as molecular determinants of endoderm fate leading to expression of squamous programs. Many of the usual mutations present in PDAC also occur in ASCP. Like PDAC, ASCP is known to have a dense and prolific desmoplastic stroma that is enriched in immunosuppressive immune cells. *Right*- Pancreatic acinar cell carcinoma (PACC) is not defined by changes in single genes. Mutational signatures suggest that impairment of the DNA damage repair system is frequent in this tumor type. Methylation and copy number changes cause downregulation of several tumor suppressors at the protein level. Activating *BRAF/RAF1* mutations and fusion products have been frequently observed. The tumor is highly cellular with little stroma, extensive vasculature, and limited immune cell infiltration. Created with BioRender.com.

The genomics of ASCP have been assessed by several groups and are summarized in Figure 2. An initial study by Brody *et al.* analyzed samples from 8 ASCP patients and identified *KRAS* codon 12 mutations in all samples, as well as frequent *DPC4* and *TP53* alterations^[53]^. Subsequently, others also demonstrated KRAS mutation in 100% of 33 combined tumor samples, making the prevalence of this mutation even higher than that seen in PDAC^[60,61]^. In addition, mutation of *TP53*, the second most frequently mutated gene in PDAC, was found in 88% of 17 ASCP cases^[61]^. Frequent amplification of the *MYC* oncogene was also noted^[62]^. Through the use of laser capture microdissection to separately isolate adenomatous and squamous components of ASCP, it was shown that both histotypes have similar genomic changes, consistent with both components deriving from a common progenitor^[61]^. This was also seen in an ASCP arising from intraductal papillary mucinous neoplasm^[63]^. In summary, the genomics of both the squamous and glandular components of ASCP closely resemble what is typical for PDAC. Novel mutations unique to ASCP have been difficult to identify. Liu *et al.* reported frequent somatic mutations in the *UPF1* RNA surveillance gene of ASCP tumors and proposed this could be a unique genetic driver of ASCP^[64]^; however, three succeeding studies in other ASCP cohorts were unable to replicate these findings^[39,61,65]^. It was later noted that 45% of *UPF1* mutations purportedly present in ASCPs were identical to normal genetic variants, and functional studies of these mutations identified no pathogenic characteristics^[66]^. Taken together, these findings suggested that ASCP and PDAC are genetically similar and may represent the same tumor type.

ASCP has numerous similarities to the basal/squamous transcriptomic subtype of PDAC^[35]^. Characteristic gene programs found in basal-type PDAC include MYC pathway activation, upregulated expression of transcription factor *TP63ΔN* and target genes, and activated EGF and TGF-β signaling. The basal/squamous subtype was also associated with epigenetic downregulation of genes involved in pancreatic endodermal cell fate determination, such as *GATA6*. Hayashi *et al*. extended these observations in their integrated analysis of the histologic, genomic, and transcriptional characteristics of PDAC^[39]^. A new classification system was developed, identifying PDAC tumors with > 30% keratinization or immunohistochemical labeling with squamous markers p63 or CK5/6 as having squamous differentiation (SD), while those tumors with some, but ≤ 30%, of cancer cells having these characteristics were termed PDAC with squamoid features (SF). Overall, 15.7% of 123 exocrine pancreatic cancer cases showed SF or SD, while 5.7% of cases met the pathologic definition for ASCP. SF/SD tumor samples, whether pathologically defined as ASCP or not, overlaid with the basal transcriptomic subtype of PDAC, meaning that as many as 10% of PDAC cases contain squamous components and have a transcriptomic profile indistinguishable from ASCP. *MYC* amplification was much more frequent in SF/SD/ASCP tumors, and *MYC* copy number was highest in areas of SF/SD within tumors heterogeneous for squamous and glandular components, although it was noted that overexpression of MYC in glandular-type PDAC did not induce squamous histology. *MYC* is a major instigator of metastasis in PDAC. *MYC* expression has also been shown to drive glycolysis and to stimulate recruitment of immunosuppressive cell types to the tumor microenvironment^[67–71]^. Of note, SF/SD tumors had a higher likelihood of mutation in a chromatin modifying gene than tumors lacking SF/SD, and the presence of these changes in conjunction with *MYC* amplification or overexpression were associated with expression of squamous differentiation markers. It is important to note that mutations in chromatin modifying genes did not exceed 50% of SF/SD cases, meaning that other genetic or epigenetic changes that lead to squamous marker expression must exist^[39]^.

### Epigenetic changes cause squamous trans-differentiation and a more aggressive phenotype

The squamous program is initiated in pancreatic cancer as in other squamous tumors by upregulation of transcription factor *TP63*. Specifically, expression of the ΔN isoform of *TP63* (*TP63ΔN)* is sufficient to drive the squamous differentiation program in PDAC; forced expression of *Tp63ΔN* in mice resulted in more aggressive tumors that grew faster, were more motile and invasive and metastasized more frequently^[72,73]^.

The upstream genetic and epigenetic changes required to stimulate *TP63ΔN* expression are slowly becoming clearer. Upregulation of *TP63ΔN* can result from inactivating mutation or loss of the histone demethylase *KDM6A*, a genomic change that has been observed in ASCP and PDAC with SF/SD^[74]^. Recently, it was shown that loss of *HNF1A*, a homeodomain transcriptional regulator that recruits KDM6A to genomic binding sites, can phenocopy *KDM6A* loss and produce a sarcomatoid tumor morphology in conjunction with *Kras* mutation in mouse pancreas^[75]^. Trans-differentiation to a squamous morphology appears to require concomitant loss of endodermal cell fate determinants such as *GATA6*, although the loss of *GATA6* alone is insufficient to drive the squamous program^[76]^. It is important to note that *KDM6A* loss is not ubiquitous in squamous trans-differentiated pancreatic cancers, and therefore one might hypothesize that other chromatin regulatory genes found to be mutated in ASCP and PDAC with SF/SD (such as *ARID1A*, *KMT2C*, *KMT2D*, *SMARCA2*, *ARID2*, *ASXL2*, *TET1* and *MSL2*^[39,62]^) may also lead to upregulation of *TP63ΔN*.

#### Summary of ASCP histology and molecular pathology

In conclusion, ASCP is an extreme form of the basal/squamous subtype of PDAC that contains both glandular and squamous regions. Squamous differentiation is primarily driven by epigenetic reprogramming through loss of chromatin regulatory elements and endodermal cell fate determinants, with consequent activation of TP63 and upregulation of MYC.

### Epidemiology and prognosis

ASCP comprises 0.38%-10% of exocrine pancreatic cancers and appears to have a slight male predominance^[49,60,77]^. ASCP is typically considered more aggressive than standard PDAC. Multiple registry studies that have evaluated large cohorts of patients concur that ASCP patients have similar demographic characteristics to PDAC patients, but present with larger and more poorly differentiated tumors^[78–81]^. Median survival in the unselected population is similar between ASCP and PDAC patients and is ~4-6 months [Table 2]. In patients who undergo resection for local or locoregional disease, those with ASCP have a lower median overall survival (OS), but similar long-term outcomes, with less than 20% of patients surviving 5 years or more [Table 3]. Adjusting for the more aggressive clinical characteristics of ASCP at diagnosis did not change the poorer outcome in two studies^[79,80]^, but matching ASCP patients to similar PDAC patients using a nested case-control design did find that survival differences in resected patients resolved^[78]^.

Recently, a nomogram utilizing 9 independent clinical and demographic risk factors was found to be predictive of overall survival and cancer-specific survival in ASCP patients^[82]^. Protective factors for increased overall survival included resection, radiotherapy, chemotherapy, negative lymph node involvement, smaller tumor size, multiple tumors, localized tumors, and being married. Female sex was also associated with prolonged cancer-specific survival. In the metastatic/recurrent disease setting, presence of lung or peritoneal metastases, anemia, high serum C-reactive protein, and CA 19-9 ≥ 1,000 conferred poorer prognosis in patients receiving chemotherapy^[83]^. These factors are largely consistent with those identified as pro-survival determinants in other studies.

### Diagnosis and imaging

Pathological diagnosis of ASCP is most straightforward in resected patients where the large quantity of tissue available allows for more conclusive determination of the percentage of squamous component in the tumor. Unfortunately, the majority of patients with ASCP do not present with resectable disease and diagnosis must be made from fine needle aspiration (FNA) or core needle biopsy. Diagnosis using these methods is possible^[84]^; however, definitive ASCP diagnosis is more difficult as patients with some squamous component noted in the sample may be diagnosed with PDAC due to the pathologist’s inability to conclusively quantitate a ≤ 30% percentage of squamous component. Note in Table 3 that ASCP constitutes at least 1% of diagnoses in resected patients, while Table 2 shows that this percentage is much lower in the full populations which include non-resected patients, although the incidence of metastatic ASCP is anticipated to be higher given its more aggressive behavior. These data suggest that the percentage of patients with ASCP in the metastatic exocrine pancreatic cancer population is likely underestimated and may be due to limitations of diagnosing ASCP from non-surgical samples.

On CT imaging, ASCP is more likely to be round or lobulated than PDAC, to have extensive central necrosis and to have more frequent accompanying portal vein thrombosis^[85]^. Indeed, central necrosis may be a predominant feature, with at least 75% of cases exhibiting this characteristic^[86]^, although others have found more evidence of ring enhancement than widespread necrosis^[87,88]^. MRI was found to be more sensitive and specific than CT for detecting ring enhancement and necrosis, and concurrence of both findings is indicative of ASCP^[88]^. Ren *et al*. have developed a novel radiomics signature that can preoperatively differentiate between ASCP and PDAC with a positive predictive value of 92% and a negative predictive value of 98%^[89]^. If this signature could be accurately applied in the non-operative setting, it might increase diagnostic accuracy for ASCP when used in conjunction with pathologic review of needle biopsy specimens.

### Clinical manifestations

ASCP patients have been reported to present with anorexia, weight loss, abdominal pain, nausea and vomiting, fever, and occasionally jaundice^[90–96]^. Jaundice may be less prominent given that primary tumors in the head of the pancreas are less frequent than seen in PDAC, with a higher incidence of body and tail tumors observed. While malignant hypercalcemia is almost never seen in PDAC, multiple cases of hypercalcemia due to elevated PTHrP (Parathyroid hormone-related protein) have been reported in ASCP patients^[97–99]^. This is likely driven by the squamous component, given that malignant hypercalcemia is not infrequently seen in other squamous solid tumors that arise from lung, esophagus, and head and neck^[100]^.

The PDAC serum tumor marker Carbohydrate Antigen 19-9 (CA 19-9) is frequently expressed in ASCP and can be used as surrogate marker of tumor response similar to PDAC^[45]^. CEA elevation can also be seen.

### Clinical management of ASCP

There are currently no treatment guidelines for ASCP. All treatment is extrapolated from standards of care for PDAC as no prospective or randomized trials examining treatment paradigms have been reported. Staging of ASCP is as per PDAC staging [Table 1].

#### Resectable disease

Resection is recommended for patients with early-stage disease and several population-based studies have demonstrated a significant survival advantage for patients with locoregional disease who undergo surgery versus those who do not^[79–81]^. R1 resection was associated with worse outcomes in one smaller single-institution study that included only one patient with this resection status^[50]^, but no contribution of R0 status to survival was found in two larger population-based studies^[78,80]^. A separate retrospective single-institution study found that a positive resection margin was negatively associated with survival^[101]^. Unfortunately, margin positive rates exceeded 20% in one study examining ASCP cases in the National Cancer Database and another large surgical case series^[49]^. Although better tumor removal during surgery appears advantageous to patients, the risks of surgery remain significant. The post-operative mortality rate at 90 days reached 6.5% for ASCP patients in one population-based study. Higher mortality was associated with more advanced age and increasing co-morbidities^[102]^. Surgery is an important part of treatment for patients with resectable disease.

Addition of chemotherapy before or after surgery appears to be beneficial. Recent retrospective population-based studies have shown improved survival outcomes in patients who receive adjuvant chemotherapy, with a > 50% decrease in mortality as compared to surgery alone. However, patients receiving multi-modal therapy were more likely to be younger and to have fewer co-morbidities than those who did not receive post-operative chemotherapy, confounding survival assessments^[80,102]^. Nevertheless, chemotherapy does appear active in ASCP patients: patients with resectable disease who received chemotherapy alone had similar median OS as those who received surgery alone, suggesting it has a similar magnitude of benefit^[102]^. In a separate, but smaller, single institution, retrospective case series where the populations receiving or not receiving adjuvant therapy were similar, receipt of adjuvant therapy was still strongly associated with improved survival. Backbones of the administered regimens were either fluoropyrimidine or gemcitabine; however, only inclusion of a platinum chemotherapy (cisplatin or oxaliplatin) improved survival in this population^[101]^. Notably, delivery of chemotherapy in the neoadjuvant versus adjuvant setting did not influence overall survival^[102]^. This is difficult to understand, given that at least 50% of pancreatic cancer patients are unable to complete their planned adjuvant therapy^[102–104]^. The optimal timing and absolute benefit of chemotherapy in resectable ASCP patients are still unclear.

#### Radiotherapy

Radiation monotherapy is associated with poor survival. Amongst all treatment modalities, using radiotherapy alone to treat ASCP patients resulted in the shortest median survival, estimated at just 2.3 months^[102]^. The relative insensitivity of ASCP to radiation therapy is somewhat surprising given that true squamous cell cancers are well known to have a higher sensitivity to radiation than adenocarcinoma and radiation can be used as definitive treatment in some clinical circumstances^[105–108]^. Although the population offered radiation alone may have been less fit than those offered other modalities confounding an accurate assessment of the absolute benefit of radiation, it is still clear that radiation will not serve as a potentially curative treatment in ASCP. Even so, neoadjuvant chemoradiation was associated with improved survival as compared to observation following resection or adjuvant chemotherapy alone and therefore should have a role in managing this stage of disease^[49]^.

The use of intraoperative radiation therapy has been documented, but data is limited to a few case studies and small case series. One such case utilized a multidisciplinary treatment approach which included upfront resection with intraoperative radiotherapy followed by adjuvant chemotherapy and radiotherapy. The reported patient enjoyed a 40-month survival^[109]^. A separate case series also documented the use of intraoperative radiation in two patients with death reported 44 and 22 months post-diagnosis^[110]^. Intraoperative radiation remains a rare treatment in this and other pancreatic diseases.

#### Use of chemotherapy in advanced disease

Palliative chemotherapy is typically given to patients with locally advanced or metastatic disease. Significant questions remain regarding what treatment regimen is best for ASCP patients. There are 2 retrospective multicenter studies and 3 case reports that have documented the response of advanced ASCP to chemotherapy. Yoshida *et al*. examined the outcomes of 116 patients with recurrent or metastatic ASCP who received chemotherapy at 24 Japanese institutions from 2001-2017^[83]^. Those receiving combination chemotherapy regimens (*n* = 57) had a trend of increased survival compared to those receiving monotherapy (*n* = 59) and a significantly higher disease control rate, and clinical characteristics were well-balanced between the cohorts. This study was large enough that response to PDAC standards GnP and FOLFIRINOX could be compared. No difference in median OS, PFS or other clinical parameters of response was found in the 28 patients receiving GnP versus the 10 treated with FOLFIRINOX, and only 5 patients receiving these treatments survived 18 months or longer. Similarly, a different retrospective multicenter study reported individual outcomes of 16 ASCP patients receiving chemotherapy for advanced disease and identified no statistical or anecdotal evidence for the superiority of a specific regimen^[111]^. Two case reports of patients with advanced disease describe rapid disease progression on standard PDAC regimens, but a third documents a patient achieving a partial response to 5-FU given in combination with cytokines IFNα and TNFα that allowed for subsequent surgical resection^[90,91,112]^. There are no reports of common regimens given for pure squamous cell cancer, such as platinum with taxane being tested in this tumor type. Current data are unclear on what constitutes an optimal chemotherapy regimen for patients with advanced ASCP.

### Clinical research

Three clinical trials specific for advanced, previously treated ASCP are currently enrolling; these are the first prospective clinical trials specific for this tumor type [Table 4]. Sequencing of several ASCPs identified FGFR activation as a lesion that is frequently present in ASCP, and at least one organoid model bearing FGFR fusion was found to be sensitive to FGFR inhibition with a pharmacologic agent^[62]^. Based upon this, a Phase 2 study of the anti-FGFR drug pemigatinib was recently initiated (NCT05216120). ASCP is now well understood to have strong overexpression/amplification of *MYC*. It was recently found that triptolide, the active ingredient of a Chinese herbal remedy, exhibits anti-cancer efficacy, at least in part, through inhibition of superenhancer complexes that drive expression of oncogenes like *MYC*. Treatment with the triptolide pro-drug minnelide can inhibit activity of *MYC* and other oncogenic superenhancers^[113]^. Based upon this data, we have initiated a Phase 2 study of Minnelide for ASCP patients, which is currently in recruitment (NCT04896073)^[114]^. ASCP has been noted to have expression of PD-L1 in the squamous components and a more tumor inflamed phenotype than standard PDAC^[51,57,58]^. Another Phase 2 study (NCT05216120) is testing the anti-PD-1 antibody retifanlimab (INCMGA00012) in ASCP patients with hopes that this tumor type will be more responsive to immunotherapy than PDAC. Results from these studies of targeted therapies are anxiously awaited.

Several rationally designed Phase 2 clinical trials of targeted agents are currently recruiting patients with these rare exocrine tumor types.

## PANCREATIC ACINAR CELL CARCINOMA (PACC)

### Pathologic characteristics

**Figure 3:**
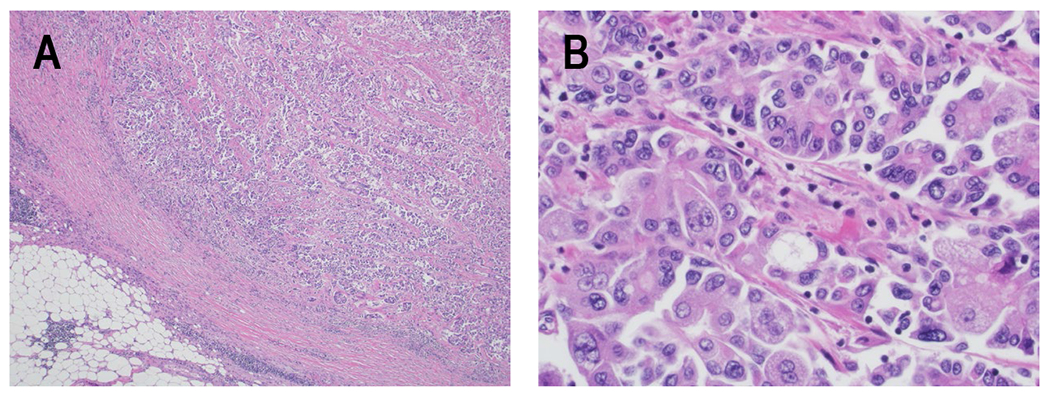
Histologic appearance of PACC. H&E specimens of PACC. A) Low power image showing how PACC recapitulates the architecture of the exocrine pancreatic parenchyma with back-to-back acinar structures containing lumina. The tumor clusters are irregular in size and shape and are approaching the peri-pancreatic adipose tissue (40x). B) Higher power image showing the classic cytologic features of PACC with basally located nuclei containing single prominent nucleoli, and abundant apical amphophilic and granular cytoplasm (400x).

Grossly, PACC appears as a well-circumscribed, lobular, tan or pink mass emerging from pancreatic acini. Hemorrhage and necrosis are frequently present as the tumor is well-vascularized. Mucin production is not seen in PACC. Microscopically, PACC is a highly cellular tumor with no desmoplasia and limited stromal elements. Well-to-moderately differentiated cases appear as nests of pyramidal-shaped cells that cluster around a lumen, the so-called *acinar* pattern^[115]^ [Figure 3]. These PACC cells have abundant eosinophilic cytoplasm that contains frequent, fine apical zymogen granules, basally located and relatively uniform nuclei, and a prominent nucleolus. As the tumor progresses, the acinar differentiation can become less pronounced, and nuclei may lose their basal orientation. Sheets of cells lacking obvious acinar structures may be present in poorly-differentiated tumors and some tumors can develop a *solid* or *trabecular* pattern with large rows of cells^[116]^. Sometimes the acinar pattern can be confused with neuroendocrine tumor rosettes and mixed acinar-neuroendocrine tumors also exist^[117]^. The absence of squamoid corpuscles differentiates PACC from pancreatoblastoma^[118]^. Immunohistochemistry and special stains can help to distinguish PACC from other tumor types. Positive staining for lipase, trypsin, chymotrypsin, and/or BCL10 is considered definitive for PACC diagnosis^[115,119]^. Trypsin and chymotrypsin together are the most sensitive markers of acinar differentiation and are positive in over 95% of cases^[120]^. Anti-BCL10 and carboxyl ester lipase are additional, highly sensitive markers^[119,121,122]^. Recently, staining for CPA1 (carboxypeptidase A1) and REG1α (lithostathine-1-alpha) were found to have excellent sensitivity and specificity for PACC, essentially excluding the diagnosis if the tumor does not stain positively^[123,124]^. PACC is morphologically and histologically distinct from PDAC.

#### Molecular profiling of PACC

Multiple studies have examined the molecular profile of PACC and identified actionable genetic changes in this tumor type^[125–127]^. Findings are summarized in Figure 2. From the first series examining whole exome sequencing of PACC, it was apparent that PACC had higher somatic mutation rates than PDAC and a high frequency of large chromosomal changes, mostly unaccompanied by mutations or methylation in known DNA strand break repair genes^[125]^. In fact, no genes were mutated at a frequency higher than 30% of cases^[125,127]^. Some genes which had somatic mutations or rearrangements in more than 1 case were: *APC, PTEN, ARID1A, SMAD4, JAK*, and *BRAF*. One series identified *BRAF* or *RAF1* rearrangements in 23% of cases and determined that at least some of these are sensitive to pharmacologic MEK inhibition^[126,128]^. Other studies have also reported *BRAF* gene arrangements in PACC^[129,130]^; however, a subsequent study could not replicate the high incidence of this finding in a larger cohort^[127]^. Cases of *RET* gene rearrangements have also been reported and these may be susceptible to RET-directed therapy^[131]^. One case of *NTRK* fusion sensitive to larotrectinib has been described^[132]^. Overall, recurrent point mutations, rearrangements or fusions of a specific gene do not appear to define this disease. Interestingly, ~10% of PACC tests positive for MSI-H/dMMR (8 of 72 tested in^[118,125,133]^) and could potentially be treated with anti-PD-1 therapy.

Moreover, analysis of mutational signatures revealed that 14 and 15 of 22 PACC cases displayed signatures related to tobacco or associated with defective DNA repair, respectively^[127]^. Inactivating genetic changes in DNA repair genes that might explain the latter signature were found at high incidence in two studies^[126,128]^, and changes in *BRCA1/2* may be particularly frequent^[134]^. However, this finding was not replicated in two other studies^[125,127]^. Uniquely, Jakel *et al*. discovered that expression of specific tumor suppressors is frequently lost in PACC through copy number aberrations and changes in methylation^[127]^. These include *ARID1A, APC, CDKN2A* and *ID3*. Downregulation of *ID3* at the protein level was nearly ubiquitous. Interestingly, this protein has been linked to DNA repair processes through a novel mechanism and to sensitivity to PARP inhibitors^[135–137]^. Actionable genetic mutations in PACC are not uncommon, such that mutational analysis of these tumors may be clinically useful in the majority of PACC patients.

It is important to note that the tumor samples used for larger -omics studies are all derived from surgical specimens, presumably representing early-stage disease exclusively. It is unclear whether similar patterns of genetic changes occur in advanced diseases. One study specifically examining *TP53* mutation in PACC did include specimens from patients with metastatic disease, and matched primary tumor specimens were available for 10 cases^[138]^. It was noted that the rate of deleterious *TP53* mutations was higher in metastatic specimens, but that the corresponding primary tumors demonstrated the same mutational profile. Moreover, in this in-depth analysis incorporating focused mutational analysis, FISH, methylation analysis and immunohistochemistry, 44% of cases contained at least one *TP53* alteration that was anticipated to affect gene function. Despite this, survival only correlated with true *TP53* loss (which occurred in only 5 of 43 cases) and was not impacted by *TP53* mutation alone. It was concluded that *TP53* loss may define a subset of more aggressive PACC tumors that have high rates of metastasis.

#### Summary of PACC anatomic and molecular pathology

PACC is not similar to PDAC at the anatomic or genetic level. It is highly cellular, never desmoplastic, and most often lacks *KRAS* mutation. The frequency of actionable gene mutation appears high, but these do not seem to cluster to a single gene.

### Epidemiology and prognosis of PACC

PACC is an extremely rare tumor; it represents just 0.2%-2% of all pancreatic malignancies in adults^[139]^. In children, PACC accounts for up to 15% of pancreatic tumors and has some clinical and morphological overlap with pancreatoblastoma^[119]^. Adult patients present with PACC about a decade earlier on average than the median age for diagnosis of PDAC. Males are more commonly affected; the male to female ratio is 2:1^[140–143]^. The reason for the male predominance is unknown. Interestingly, clinical outcomes are superior in females with PACC^[143]^. Retrospective population-based studies suggest that 32%-54% of patients present with distant metastatic disease^[142–145]^. The most frequent site of metastasis is the liver. While this proportion is similar (in some studies) to that for PDAC, PACC patients have improved median survival compared to PDAC at every disease stage^[145]^. This is particularly notable in patients with PACC who undergo resection, where median OS can exceed 70 months, but rarely reaches 36 months in PDAC patients. PACC tumors are typically larger at diagnosis than those seen in PDAC. Interestingly, primary tumor size did not correlate with the time to onset of metastatic disease or with the presence of metastatic lesions^[141]^. Those with mixed acinar-neuroendocrine and acinar-ductal pathologies appear to have similar prognosis and tumor characteristics^[146]^. PACC is still an aggressive tumor, but prognosis is superior to PDAC. PACC retrospective case series examining clinical outcomes are summarized in Table 5 ^[142–148]^.

PACC has been seen in kindreds with familial adenomatous polyposis^[149]^, Lynch syndrome^[150]^, and *BRCA* mutation^[134,151]^, but it is difficult to determine how influential these genetic syndromes are in this disease given the small number of cases overall. Specific clinical or environmental risk factors for PACC remain unknown.

### PACC diagnosis and imaging

Diagnosis of PACC is by pathology. Tumor samples can be obtained through surgical resection or needle aspiration. Diagnosing PACC from a fine needle aspiration can present a challenge as cytologic samples can be easily confused with normal acinar cells or sampling from a neuroendocrine tumor. Labate *et al*. were the first to describe the cytopathology of PACC in detail and were able to correctly diagnose PACC from a cytology specimen in just 2 of 7 cases^[152]^. Subsequently, the diagnostic yield has not appeared to improve, as only 2 of 7 cases reported since could be definitively diagnosed as PACC based on cytology alone^[153–156]^.

CT is the standard and first line choice for imaging studies of PACC. Visible lymph nodes were noted to be more frequent in PACC than PDAC. Tumor hypoattenuation in arterial phase compared to the uninvolved pancreas was much less frequent in PACC and PACC was much more likely to have well-defined margins than PDAC. Lack of bile duct dilatation was also much more common in PACC than PDAC^[157]^. MRI characteristics of PACC were investigated in a small study of 5 patients, and it was concluded that PACC presents as a round, encapsulated tumor with moderate and heterogeneous enhancement after gadolinium administration^[158]^. Either CT or MRI can adequately detect PACC.

### Clinical manifestations of PACC

The most common symptoms in newly diagnosed PACC patients are generally non-specific: abdominal pain and weight loss occur most frequently^[141]^. Jaundice is an infrequent symptom even when PACC presents in the pancreatic head. Jaundice is very common in PDAC and PACC tumors are typically larger at diagnosis^[141,148,159]^, so the rarity of jaundice in PACC has been difficult to understand. It has been suggested that the more encapsulated biology of PACC is less susceptible to causing jaundice than the more infiltrative biology of PDAC.

Lipase hypersecretion syndrome (LHS) is a paraneoplastic syndrome uniquely observed with PACC. Many PACCs produce lipase and excessive release of this enzyme into the circulation occurs in about 10%-15% of PACC patients, leading to accumulation of pancreatic enzymes in peripheral tissues and subsequent enzymatic digestion. Very elevated blood lipase levels can cause eosinophilia and panniculitis that most often manifests as painful subcutaneous nodules that resemble erythema nodosum. Panniculitis of LHS has a predilection for pressure points in the lower extremities, especially on the shins^[160,161]^. Another common feature of LHS is painful and inflammatory polyarthritis that resembles rheumatic fever or gout. It is thought to be secondary to periarticular fat necrosis. Joint aspirates are typically sterile but occasionally crystals are visualized^[162,163]^. Patients experiencing LHS usually have advanced metastatic disease. LHS in PACC has been recently well-reviewed by Taskin *et al*.^[164]^.

Levels of the PDAC serum tumor marker CA 19-9 are elevated in less than 30% of patients with PACC and may be related to non-specific biliary duct irritation rather than tumor production in most cases^[141]^. Nevertheless, poor survival outcomes were observed in patients with tumors that do express CA 19-9^[146,147]^. Larger cohorts would be required to assess the prognostic value of this marker in PACC. Elevation of CEA has been observed in about one-fifth of PACC patients^[141]^, and elevated blood Alpha-fetoprotein (AFP) levels in younger patients and should raise suspicion of PACC, especially when associated with a pancreatic mass^[165]^. Serum lipase might be used as a surrogate marker of tumor response in some patients, as PACC frequently produces high levels of lipase, but this has not been well characterized in the literature.

### Clinical management of PACC

There are currently no treatment guidelines for PACC. All treatment is extrapolated from standards of care for PDAC as no prospective or randomized trials examining treatment paradigms have been reported. Staging of PACC is as per PDAC staging [Table 1].

#### Resectable disease

Surgical resection is the treatment of choice for early-stage disease. Resection has been shown to result in a dramatic improvement in cohort overall survival as compared to no resection in multiple retrospective case series and population-based studies^[141–144,166]^. In fact, resection was the most predictive factor for overall survival in some studies. In one study, 5-year survival for Stage I or II patients receiving resection was 42% compared to 9% for those who did not get surgery^[167]^. Negative margins are predictive of better long-term survival in some studies.

Recurrence following surgery is estimated to be > 50%, especially in patients with Stage II or III disease; however, it remains unclear whether addition of adjuvant therapy is of benefit to PACC patients. Patients receiving adjuvant therapy had better OS than those that did not in one large retrospective population-based study^[142]^. Others found that the benefit was only associated with administration of platinum-based chemotherapy^[141]^ or presence of node-positive disease^[166,168]^. A fourth study found no survival benefit for patients who received adjuvant chemotherapy compared to those who did not^[143]^. Choice of chemotherapy regimen may prove a confounder in the latter study; single-agent gemcitabine was the chemotherapy regimen administered to 60% of patients in that study, and as discussed below, the benefit of gemcitabine in PACC appears to be limited. Another smaller study of 9 patients found that patients receiving 5-FU-based adjuvant chemotherapy had superior overall survival to those receiving a gemcitabine-based regimen^[169]^. A randomized study of adjuvant therapy in PACC could resolve this question but is unlikely to be feasible given the rarity of the tumor.

Neoadjuvant therapy has not been thoroughly explored in this disease. One case study reported on a 65-year-old PACC patient who had a complete pathologic response to neoadjuvant modified FOLFIRINOX and remained in remission at 33 months post-resection without adjuvant treatment. It is anticipated that more resectable PACC patients may receive neoadjuvant therapy, given the trend towards this paradigm in PDAC.

#### Recurrent disease following resection

Re-resection at the time of recurrence has been performed in selected cases of PACC with some success^[170,171]^. One case report identified a patient with recurrent disease who appeared to be cured after treatment with radiofrequency ablation (RFA) and combination chemotherapy and a second who experienced a long remission after RFA^[172]^. These case reports suggest that local therapy may still be curative in some cases of post-surgical recurrence.

#### Advanced disease

In metastatic or locally advanced PACC multiple therapeutic modalities have been used. Unlike in PDAC, metastasectomy is sometimes performed in conjunction with resection of the primary tumor. One single institution case series of 64 PACC patients found that patients with distant metastases who received surgery had no difference in overall survival compared to those who did not^[141]^, but it is unclear how many received surgery with curative intent versus a palliative procedure. Multiple case reports describe metastasectomy being beneficial in individual patients. Sumiyoshi *et al*. reported ongoing recurrence-free survival at 73 months for one patient following resection of the primary tumor in combination with removal of several synchronous peritoneal nodules followed by continuous S-1 chemotherapy^[173]^. Another case study described a patient who had multiple metastasectomies between sequential lines of combination chemotherapy that has survived over 10 years since initial diagnosis^[174]^. A third case report outlined the course of a metastatic patient who received neoadjuvant capecitabine/oxaliplatin followed by resection of the primary tumor with hepatectomy resulting in over 30 months of disease-free survival before recurrence. The patient subsequently restarted chemotherapy and remained in complete response for the last 3 years^[175]^. These inspiring cases suggest that outcomes of metastasectomy in PACC may more closely resemble those of colon cancer patients than PDAC patients and that surgical intervention could be of benefit to patients with oligometastatic disease in selected cases. Further study of this treatment paradigm is needed.

For patients with locally advanced disease, upfront FOLFIRINOX chemotherapy can be successful enough at debulking to allow resection^[176]^. Descriptions of successful debulking with other regimens have not been described.

Systemic chemotherapy is the mainstay of treatment for patients with advanced PACC. Several groups of investigators have analyzed retrospective case series to identify the most beneficial regimens for advanced PACC patients^[169,177–179]^. Overall, the objective response rate to FOLFIRINOX appears to be higher than for any other regimen, with 16 of 21 patients receiving this regimen in the first-line setting achieving objective responses^[169,178,180,181]^. All studies also agree that gemcitabine monotherapy has poor efficacy in PACC, with no responses reported in 10 patients described in 2 studies. Similarly, gemcitabine-based regimens overall are inferior to fluoropyrimidine-based regimens, with significantly lower progression-free survival observed in multiple studies^[169,178]^. Intriguingly, objective responses have been noted to single-agent infusional 5-fluorouracil, S-1 and capecitabine; however, addition of irinotecan or platinum agents clearly increases efficacy. In a study of 58 advanced disease patients, Takahashi *et al*. reported a 40% response rate to platinum-containing regimens and a 29% response rate to irinotecan-containing regimens in the first-line advanced disease setting. In addition, patients who received these regimens had improved overall survival^[177]^. One important caveat of these findings is that almost all of these case series are from Asia, and it is unclear whether similar benefits to irinotecan and fluoropyrimidine containing regimens will be reproducible in a majority Western population who metabolize these drugs differently. However, smaller case series in Western countries suggest the relationship holds true regardless of race/ethnicity.

Targeted therapies have been demonstrated to produce clinical benefits in PACC patients with sensitizing mutations. Responses to platinum-containing regimens appear particularly strong in PACC patients with *BRCA* mutation, and a beneficial treatment effect of olaparib in this setting has also been reported^[151,178]^. A complete response to the RAF/MEK inhibitor combination of dabrafenib/trametinib has been reported in 2 patients with tumors bearing *BRAF* V600E mutation^[182,183]^. Treatment with alectinib of a metastatic PACC patient with a somatic *KANK4/ALK* fusion resulted in a partial response ongoing at 1 year^[184]^. The use of immunotherapy in a PACC patient with high tumor mutational burden and tumor PD-L1 expression resulted in a partial response that permitted subsequent re-resection with continued absence of disease^[185]^. It is very clear that precision medicine is of significant benefit to PACC patients with actionable tumor genetics and providers should advocate for sequencing of available tumor material to expand treatment options.

Immunotherapy has not been extensively evaluated in PACC. MSI-H/dMMR disease is not uncommon in PACC and would be expected to respond to anti-PD-1 therapies. It is unclear whether patients lacking microsatellite instability would respond to single-agent immunotherapy treatment. However, combination of lenvatinib with an anti-PD-1 therapy was reported effective in one case study, resulting in a partial response ongoing for 21 months^[186]^. Combination of anti-PD-(L)1 therapies with anti-angiogenic agents like lenvatinib has produced exciting results in several tumor types^[187,188]^. PACC is a highly vascular tumor, like renal and hepatocellular carcinomas which have been responsive to this combination. Evaluation of this combination in PACC would be an intriguing prospect.

### Research on PACC

We have recently opened a Phase 2 study evaluating olaparib in patients with previously treated PACC (NCT05286827). To our knowledge, this is the first-ever PACC-specific prospective treatment study [Table 4]. This study is based upon the observation that over 90% of PACC patients have down-regulation of *ID3*, and that PARP inhibitor is active in ID3-deficient tumors^[127,136]^. Dedicated clinical studies of PACC are extremely difficult to accrue, given the extreme rarity of the disease.

## SUMMARY AND CONCLUSIONS

Consensus guidelines for rare exocrine pancreatic tumors like ASCP and PACC have not been published. Consequently, these tumors are most frequently treated using standards of care established for PDAC. While ASCP may represent an extreme variant of the basal transcriptomic subtype of PDAC, PACC shares few morphologic, histologic or molecular characteristics with PDAC and application of PDAC treatment paradigms to PACC is inappropriate.

Based on our review of the existing literature, it is clear that ASCP is a more aggressive tumor than PDAC and responses to current PDAC treatments are inadequate. Solving the puzzle of how best to control ASCP is likely to benefit the larger PDAC population, given the overlap of ASCP and the most aggressive subtypes of PDAC that harbor SF or SD. Research into treatment paradigms for basal-type PDAC, which is largely synonymous with PDAC with SF/SD, has expanded significantly over the last several years, but has resulted in limited clinical advances thus far. Recent studies have suggested that basal-type PDAC is less sensitive to platinum chemotherapy than classical-type^[189,190]^. Intriguingly, the available ASCP data hint at platinum chemotherapy providing benefits to this patient population. It is worth considering whether the more extensive squamous differentiated component of ASCP may have sensitivities to unique chemotherapy regimens that are less effective in pure adenocarcinoma. Perhaps more importantly for this population, new drugs targeting activated KRAS appear to be imminent and could turn the tide in this KRAS-driven disease.

PACC is a tumor that shares few characteristics with PDAC other than arising in the pancreas and preferentially metastasizing to the liver. It is clear that surgical resection of this less aggressive tumor provides significant benefit to early-stage patients, and investigation into the role of metastasectomy in treating PACC is also warranted. A benefit of adjuvant chemotherapy to cure early-stage patients is likely to present for PACC patients with lymph node involvement, but unclear in patients with more limited disease. Systemic therapy with platinum agents produces the best outcomes in PACC patients. Regimens with a fluoropyrimidine backbone are preferred in treating PACC, as the use of gemcitabine in this disease has resulted in inferior outcomes. All patients with PACC should undergo molecular testing for somatic mutations as these occur at high incidence and are frequently actionable, and treatment with matched therapy has been highly beneficial in case reports. In addition, the high rate of MSI-H/dMMR occurrence in PACC should prompt universal evaluation of these markers which could demonstrate sensitivity to immunotherapy. Given the extreme rarity of this disease, a coordinated research effort across many institutions will likely be required to generate sufficient cases to inform evidence-based clinical care for PACC.

**Figure 4:**
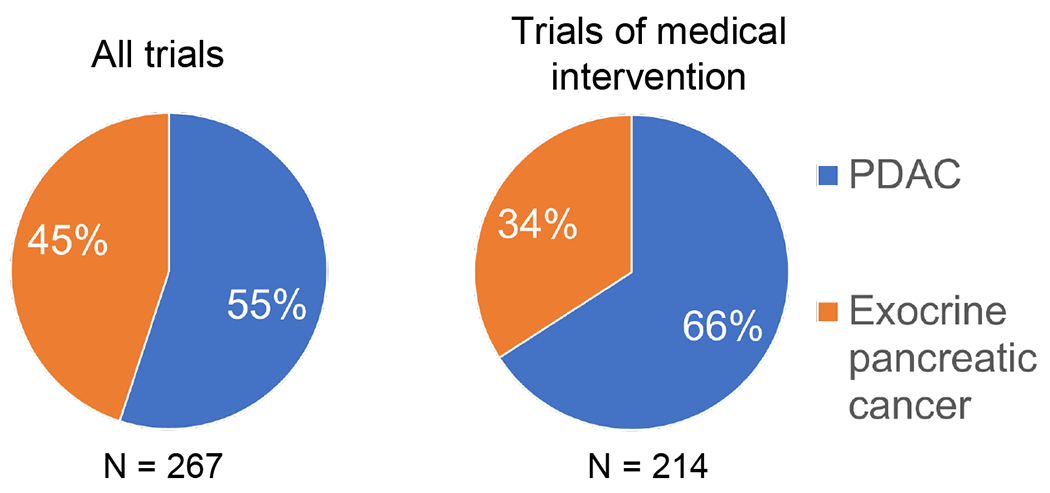
Inclusion of rare exocrine tumor patients on clinical studies of pancreatic cancer. Study registrations on clinicaltrials.gov over the last two years (from 8/2020 through 8/2022) were searched for those that were interventional, enrolled adults, and included the term *pancreas cancer*, resulting in identification of 425 records. Eliminating those specific for PNET, carcinoid syndrome or endocrine tumors resulted in 405 entries. 267 of these were specific for pancreas cancer and did not include other tumor types. After exclusion of those evaluating screening regimens, diagnostic procedures (imaging, molecular profiling), interventional procedures (endoscopy, surgery, anesthesia), and psychological, dietary, or lifestyle modification, 214 studies testing medical interventions were identified. The percentage of clinical studies specific for pancreas cancer that allow only PDAC patients versus any exocrine pancreatic cancer patient are depicted.

Dedicated clinical trials for ASCP and PACC are uncommon. These studies are difficult to accrue given the rarity of the diseases. It is also unclear how many ASCP and PACC patients understand that their pancreatic cancer is different from “standard” pancreatic cancer and would be interested in seeking more tailored treatment. We have found that at least one-third of pancreas cancer trials testing medical interventions permit the accrual of patients with any exocrine pancreatic tumor rather than limiting eligibility to PDAC [Figure 4]. While this offers benefits to patients by providing additional treatment options, these treatments may not be well justified in ASCP or PACC and are unlikely to advance our understanding of these rare diseases when it is likely that, at most, a single rare tumor patient will be enrolled, and their availability could further reduce the patient pool willing to seek out ASCP- or PACC-specific studies.

In summary, rare exocrine tumors of the pancreas present a clinical challenge since treatment guidelines are lacking. However, existing data, as described above, could form the nucleus of what constitutes best practice for these diseases.

## Figures and Tables

**Table 1.** AJCC staging for PDAC

Stage 0	Tis	N0	M0
Stage IA	T1	N0	M0
Stage IB	T2	N0	M0
Stage IIA	T3	N0	M0
Stage IIB	T1, T2, T3	N1	M0
Stage III	T1, T2, T3, T4	N2	M0
		Any	M0
Stage IV	Any	Any	M1
T	Primary Tumor		
TX	Primary tumor cannot be assessed		
T0	No evidence of primary tumor		
Tis	Carcinoma in situ, including: High-grade pancreatic intraepithelial neoplasia Intraductal papillary mucinous neoplasm with high-grade dysplasia Intraductal tubulopapillary neoplasm with high-grade dysplasia Mucinous cystic neoplasm with high-grade dysplasia		
T1	Largest tumor diameter < 2 cm		
T1a	Largest tumor diameter ≤ 0.5 cm		
T1b	Largest tumor diameter > 0.5 cm and < 1 cm		
T1c	Largest tumor diameter 1-2 cm		
T2	Largest tumor diameter > 2 cm and ≤ 4 cm		
T3	Largest tumor diameter > 4 cm		
T4	Tumor involves the celiac axis, superior mesenteric artery, and/or common hepatic artery, regardless of size		
N	Regional Lymph Nodes		
Nx	Regional lymph nodes cannot be assessed		
N0	No regional lymph node metastasis		
N1	Metastasis in 1-3 regional lymph nodes		
N2	Metastasis in ≥ 4 regional lymph nodes		
M	Distant Metastasis		
M0	No distant metastasis		
M1	Distant Metastasis		

**Table 2.** Comparison of ASCP to PDAC populations

Study	Data source	No. Of patients		Poor diff (%)		Node-positive (%)		Tumor size (cm)		Median OS (mo)		OS (%)		

		ASCP	PDAC	ASCP	PDAC	ASCP	PDAC	ASCP	PDAC	ASCP	PDAC	ASCP	PDAC	Time
[79]	SEER	415 (0.9%)	45693	*71.4*	*45*	52.8	47.1	*5.7*	*4.3*	4	5	10.8	10.9	2y
[80]	NCDB	1745 (0.8%)	205328	*40.6*	*17.3*	*21.9*	*14.8*	56%*	33.1%*	5.7	6.2	13	13.8	2y
[81]	CA Cancer Reg	95 (0.6%)	14746							4	NR			

Digits in italics indicate the study found a statistically significant difference in this measure.

* Indicates measurement of percent tumors ≥ 4 cm. Poor diff: Poorly differentiated tumor on histology; OS: overall survival; NR: not reported.

**Table 3.** Comparison of resected ASCP to PDAC

Study	Data source	No. Of patients		Poor diff (%)		Node-positive (%)		Tumor size (cm)		Median OS (mo)		OS (%)		

		ASCP	PDAC	ASCP	PDAC	ASCP	PDAC	ASCP	PDAC	ASCP	PDAC	ASCP	PDAC	Time
[78]	single institution	91 (2.3%)	3918	*79.5*	*35.9*	*88*	*78*	*4.0*	*3.2*	10.8	20.5	18.2	17.5	5y
[79]	SEER	176 (1.0%)	17411	*49.4*	*27.2*	51.4	48.4	*5.3*	*3.9*	*12*	*16*	*29*	*35.8*	2y
[80]	NCDB	503 (1.4%)	35492	*57*	*33*	*55*	*61*	53%*	30%*	*14.8*	*22*	18.2	19.2	5y
[81]	CA Cancer Reg	31 (1.5%)	2071			57.7	60.2	*4.6*	*3.3*	*12*	*NR*			

Digits in italics indicate the study found a statistically significant difference in this measure.

* Indicates measurement of percent tumors ≥ 4 cm. Poor diff: Poorly differentiated tumor on histology; OS: overall survival; NR: not reported.

**Table 4.** Clinical trials specific to ASCP and PACC

NCT#	Tumor	Stage	Agent	Mechanism	Phase
NCT04116073	ASCP	advanced	retifanlimab	anti-PD-1	2
NCT04896073	ASCP	advanced	minnelide	superenhancer inhibitor	2
NCT05216120	ASCP	advanced	pemigatinib	FGFR2 inhibitor	2
NCT05286827	PACC	previously treated	olaparib	PARP inhibitor	2

**Table 5.** Clinical outcomes in PACC

Reference	Data source	Years	n	Age at diagnosis (median)	Median tumor size (cm)	With metastasis (%)	OS (median)	Stage 4 OS (median)	Early stage OS (median)
[142]	NCDB	1985-2005	865	67	]5.9	32.1	N/A	17.2%*	NR-22.6m
[145]	SEER	1988-2003	672	56		53.1	47	22%*	72%*^
[144]	SEER	2004-2016	252	63.8	N/A	54.4	10	7	18,29^
[143]	German Cancer Registry Group	2000-2019	233	66	N/A	33.9	22	6	34^
[141]	SI-Harvard Hospitals	1996-2019	66	64	4.3	42	13	15	38
[147]	Korean Tumor Registry System	2003-2018	59	59.2	4.6	0	N/A	N/A	78.8^
[148]	SI-West China Hospital	2006-2016	52	50.8	5.0	30.8	39	N/A	48^
Seo 2017	SI-Asan Medical Center	1997-2015	20	57	4.0	0	N/A	N/A	75^
Matos 2009	MI	1998-2008	17	59	5.3	23.5	19	N/A	61^

Multiple retrospective case series examining survival in PACC patients have been conducted.

* indicates that 5-year overall survival percentage is documented;

^ indicates value is for resected patients only;

SI: Single institution; MI: multi-institution; n: number of patient cases; OS: overall survival; N/A: not available; NR: not reached.

## Data Availability

Not applicable.
